# Environmentally Friendly Photoluminescent Coatings for Corrosion Sensing

**DOI:** 10.3390/polym17030389

**Published:** 2025-01-31

**Authors:** Carmen R. Tubio, Laura Garea, Bárbara D. D. Cruz, Daniela M. Correia, Verónica de Zea Bermudez, Senentxu Lanceros-Mendez

**Affiliations:** 1 BCMaterials, Basque Center for Materials, Applications and Nanostructures, UPV/EHU Science Park, 48940 Leioa, Spain; laura.garea.mayordomo@gmail.com (L.G.); senentxu.lanceros@bcmaterials.net (S.L.-M.); 2 Centre of Chemistry, University of Minho, 4710-057 Braga, Portugal; barbaraddcruz@gmail.com (B.D.D.C.); dcorreia@quimica.uminho.pt (D.M.C.); 3 Physics Centre of Minho and Porto Universities (CF-UM-UP) and Laboratory of Physics for Materials and Emergent Technologies (LapMET), University of Minho, 4710-057 Braga, Portugal; 4 Chemistry Department and CQ-VR, University of Trás-os-Montes e Alto Douro, 5000-801 Vila Real, Portugal; vbermude@utad.pt; 5 IKERBASQUE, Basque Foundation for Science, 48009 Bilbao, Spain

**Keywords:** corrosion, photoluminescence salts, lanthanide complexes, sensing

## Abstract

Although an increasing number of studies are being devoted to the field of corrosion, with topics from protection to sensing strategies, there is still a lack of research based on environmentally eco-friendly materials, which is essential in the transition to sustainable technologies. Herein, environmentally friendly composites, based on photoluminescent salts dispersed in vegetable oil-based resins, are prepared and investigated as corrosion sensing coatings. Two salts NaA, where A- is a lanthanide complex anion (with Ln = Nd^3+^, and Yb^3+^), are incorporated into the resins as active functional fillers and different coatings are prepared on carbon steel substrates to assess their functional properties. The influence exerted by a corrosive saline solution on the morphology, structural, and photoluminescent properties of the coatings is evaluated, and their suitability for the practical detection of the early corrosion of coated carbon steel is demonstrated. The photoluminescence of the composite coatings depends on the corrosion time, with the effect being more important for the coatings doped with Nd^3+^. The present work shows that the composites obtained are suitable candidates for corrosion sensing coating applications, offering improved sustainability.

## 1. Introduction

Undesired corrosion is a relevant problem in several sectors and industries, including the energy, transport, and automotive sectors. In addition, it is relevant to infrastructure, such as bridges, pipelines, and highways, among other areas [1]. Consequently, in the past decades, extensive research has been performed on the development of efficient strategies to improve the protection, continuous monitoring, and early detection of corrosion processes. The great majority of studies related to corrosion protection have focused on organic/inorganic coatings, the removal of corrosive constituents, the use of inhibitors, and anodic/cathodic protection [2,3,4]. In parallel, during the past decade, extensive self-repairing strategies have been introduced in protective coatings [5,6]. 

In addition to the well-established approaches, the implementation of sensing capabilities is recognized as one of the most effective ways to early detect, monitor, and control corrosion processes. The implementation of sensing technologies essentially relies on the use of optical fibers [7,8], electrochemical noise detection [9,10], pH-indicating agents [11,12], and smart materials which can provide qualities of self-healing, damage sensing, and stimulus responsiveness [13,14]. In particular, fluorescence-based sensors have been proposed as a suitable sensing solution due to their high sensitivity and real-time detection [15]. Their working principle is based on the use of fluorescent indicators, which can be sensitive to pH or metal ion variations and can be added to solutions or coatings. Several reports have demonstrated that these fluorescent indicators can act as sensors, with promising results in the corrosion field [13,16]. However, one of the most critical issues is the design and synthesis of composites with environmentally friendly materials. 

In this context, many luminescent salts are environmentally friendly compounds which properties can be tailored through the judicious choice of the nature of the cations and anions. This advantage, plus the fact those materials may be incorporated into specific polymer matrices, has boosted their use in a wide range of applications [17]. Concerning luminescence characteristics, the introduction of lanthanide metal ions, such as lanthanide (Ln^3+^) ions, can yield highly luminescent compounds with narrow emission bands, photostability, long decay times, and high quantum efficiency [17,18]. The potential of luminescent salts has been exploited in various applications, such as anticounterfeiting [19,20] and biomedical imaging [21,22], among others [23,24,25]. Yet, in the field of corrosion sensing, such luminescent compounds have scarcely been explored. Therefore, taking into consideration the current pressing need for more sustainable approaches and materials, it is suitable to evaluate the use of luminescent materials as active fillers to develop practical sensors in the area of corrosion. Another important aspect is the selection of the host polymeric matrix, which should also be environmentally sustainable to reduce the use of synthetic materials and address the requirement of eco-friendly formulations towards sustainable materials, processes, and technologies. In this regard, vegetable oil-based resins have emerged as promising candidates on account of being abundantly availabl, and environmentally friendly. At present, vegetable resins are being increasingly employed in the coating industry, demonstrating potential applicability in corrosive environments [26]. 

In this work, we propose a novel approach to develop sensing materials for corrosive environments. This implies the use of a soybean-based vegetable UV-curable resin doped with Na[Ln(tta)4], where Ln = Nd or Yb and tta– is tetrakis(thenoyltrifluoroacetonate. The latter sodium salts are the precursor compounds of luminescent salts, the applicability of which has been already demonstrated in anticounterfeiting [19,20]. Herein, we demonstrate the applicability of such composite materials in corrosion sensing through the evaluation of the sensing performance in coated carbon steel substrates in saline environments. Accordingly, immersion experiments are performed in 5 wt. % NaCl solution for several days. In the present work, the coating is directly placed on top of the corroding surface, whereas in specific applications, such as very harsh environments, the sensing layer can be placed on top of another more protective layer. This work represents a basis for a new sensing approach in the area of steel corrosion, with improved performance and sustainability.

## 2. Materials and Methods

### 2.1. Materials

A transparent soybean-based and biodegradable photopolymer resin (ECO UV resin, Anycubic, Shenzhen, China) was used as received. 

2-thenoyltrifluoroacetone (Htta) (99%, Sigma-Aldrich, St. Louis, MO, USA), ethanol (EtOH) (99.8%, Fisher Chemical, Loughborough, UK), neodymium(III)chloride hexahydrate NdCl_3_·6H_2_O, 99.9%, Sigma-Aldrich, USA), ytterbium (III)chloride hexahydrate (YbCl_3_·6H_2_O, 99.9%, Sigma-Aldrich), and sodium hydroxide (NaOH, Merck, Rahway, NJ, USA) were used as received.

### 2.2. Preparation of Ln-Based Salts

**Figure 1:**
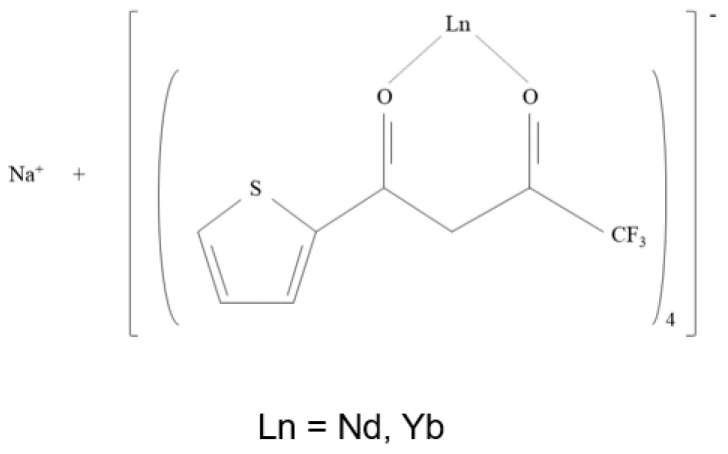
Chemical structure of Na[Ln(tta)_4_].

Two Ln ternary complexes were prepared according to the procedure reported in [19,20]. Briefly, the synthesis involved the dissolution of Htta in EtOH under magnetic stirring, followed by deprotonation with NaOH at 50–60 °C for 2 h. Then, NdCl_3_·6H_2_O, was dissolved in EtOH and was added dropwise into the previous solution. The mixture was kept at 50–60 °C for 1 h, and then the EtOH was evaporated. Afterwards, the resultant salt was dried at 50 °C for 3 days. Similarly, Na[Yb(tta)_4_] synthesis was achieved using YbCl_3_·6H_2_O as a lanthanide metal precursor. Figure 1 shows the chemical structure of Na[Ln(tta)_4_], where Ln = Nd or Yb, and tta– is tetrakis(thenoyltrifluoroacetonate.

### 2.3. Sample Preparation and Corrosion Experiments

The coating solutions were based on the vegetable UV-curable resin mixed with the sodium salts in a planetary mixer (Thinky ARE-250, Tokyo, Japan) at 2000 rpm for 4 min several times. The salt loading was controlled at 5 and 10 wt. %. Carbon steel substrates (DC01, Laser Norte S.A., Biscay, Spain) with dimensions of 5 mm × 2 mm × 0.5 mm were coated with the Ln-doped solutions using the doctor blade technique and the as-coated films were cured in a UV curing chamber (UVACUBE 400, Hönle AG, Gilching, Germany) for 3 min at 400 W. 

The influence of saline solutions on the coated steel substrates was evaluated by immersing the samples in a 5 wt. % NaCl solution. This study was carried out for 4 days, and the results were evaluated on days 1 and 4. The nomenclature adopted to represent the prepared samples was resin-xLn (with x%wt.).

### 2.4. Characterization of the Samples

The Fourier Transform Infrared (FTIR) spectra were recorded on a Jasco FT/IR-6100 equipped with an Attenuated Total Reflection (ATR) accessory (Easton, MD, USA). The samples were scanned in the 600–4000 cm^−1^ spectral range with a 4 cm^−1^ resolution.

The morphology and elemental compositions of the samples were observed by a scanning electron microscope (SEM, Carl Zeiss EVO-40, Berlin, Germany) equipped with an energy-dispersive mode (EDX, Oxford Instruments, Abingdon, UK). Before the analysis, the samples were sputtered with a 10 nm thin gold layer. 

Energy-dispersive X-ray fluorescence (ED-XRF) analysis was performed using the Spectro Midex SD system (Ametek, Inc, Leicester, UK). 

Photoluminescence (PL) spectra were recorded using a fluorescence spectrometer (FLS980, Edinburgh Instruments Ltd., Livingston, UK) with a Xenon lamp 450 W. The excitation wavelength was 300 nm.

## 3. Results

### 3.1. Active Coating Characterization

**Figure 2:**
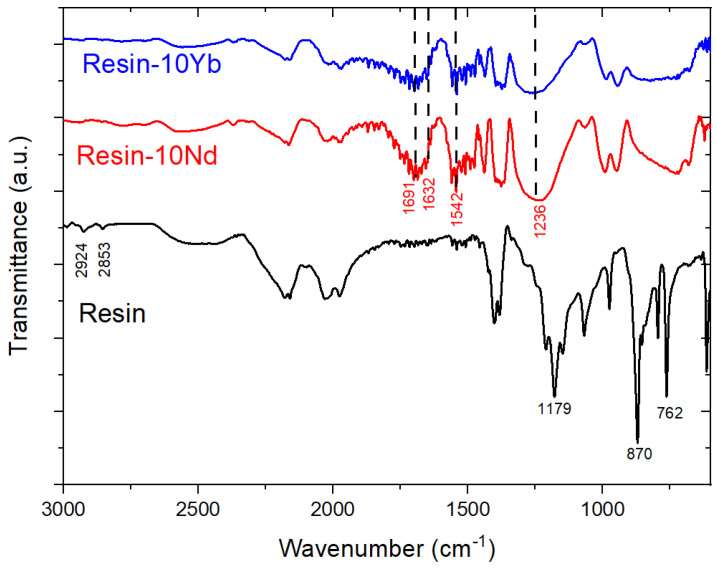
FTIR spectra of the neat resin and resin-10Ln samples.

FTIR analysis was carried out to evaluate the nature of the interactions between the UV-curable resin matrix and the Na[Ln(tta)_4_] salts. The FTIR spectra of the neat resin and resin-10Ln (Ln = Nd and Yb) samples in the 3000–600 cm^−1^ wavenumber range are shown in Figure 2.

The FTIR signature of the doped samples was markedly different from that of the neat resin. The FTIR spectrum of the pristine resin showed the typical bands of soybean oil [27,28,29]. In particular, the characteristic bands of the C-O bond were observed at 1179 cm^−1^, as were the bands at 2924 and 2853 cm^−1^. This was related to the presence of the weak asymmetric stretching of the -C-H bond. In addition, the bands of higher intensity at 870 and 762 cm^−1^ allowed us to identify the =C-H bond bending mode. On the other hand, with the presence of the Ln-based salts, new peaks were identified in the composites. These were related to the presence of the filler. In particular, bands that were specific to the β-diketonate ligand were identified at 1542 cm^−1^ due to the presence of the C=C bond, at 1691 and 1632 cm^−1^, which was ascribed to the carbonyl (C=O) group, and at 1236 cm^−1^, which was associated with the C-CF3 group [30]. No new bands or energy shifts of the resin and Ln-based salts bands were observed in the composites, indicating a lack of strong interactions between the different composite components. 

**Figure 3:**
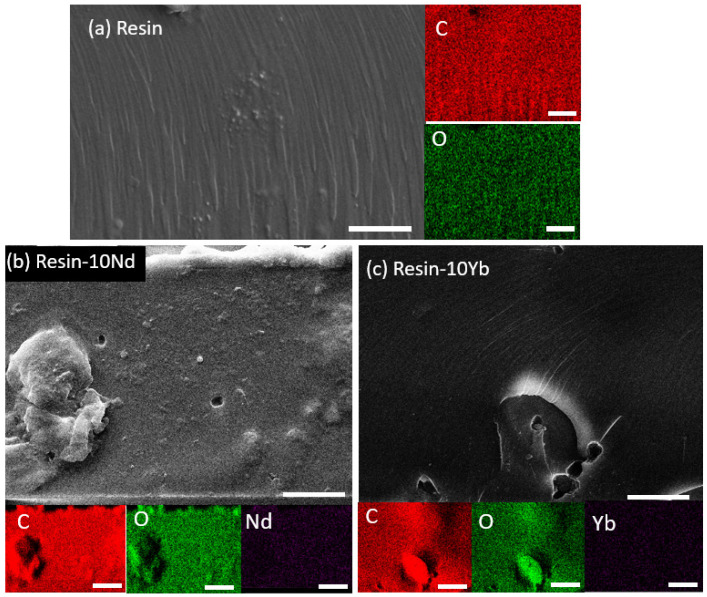
Cross-sectional SEM image and corresponding EDS elemental mapping of the resin-xLn as-prepared samples: (**a**) neat resin; (**b**) resin-10Nd; (**c**) resin-10Yb. Scale bar: 10 µm.

To study the morphology and microstructure of the samples, SEM and EDX measurements were performed (Figure 3). Figure 3a shows a representative SEM image taken from the cross-sectional area of the neat resin, and the corresponding EDX mapping of the C and O elements. The SEM image reveals that the as-prepared sample is characterized by a smooth and compact morphology, where C and O are homogeneously distributed all along the polymer. The relative distribution of these elements was in agreement with the composition of the vegetable resin made from soybean oil. Furthermore, as observed in Figure 3b,c, the dense morphology was maintained after salt addition and the corresponding EDS map demonstrates the uniform distribution of C, O, Nd, and Yb within the resin matrix, i.e., the excellent distribution of the filler within the polymer matrix, with no large aggregates or voids.

### 3.2. Corrosion Tests

**Figure 4:**
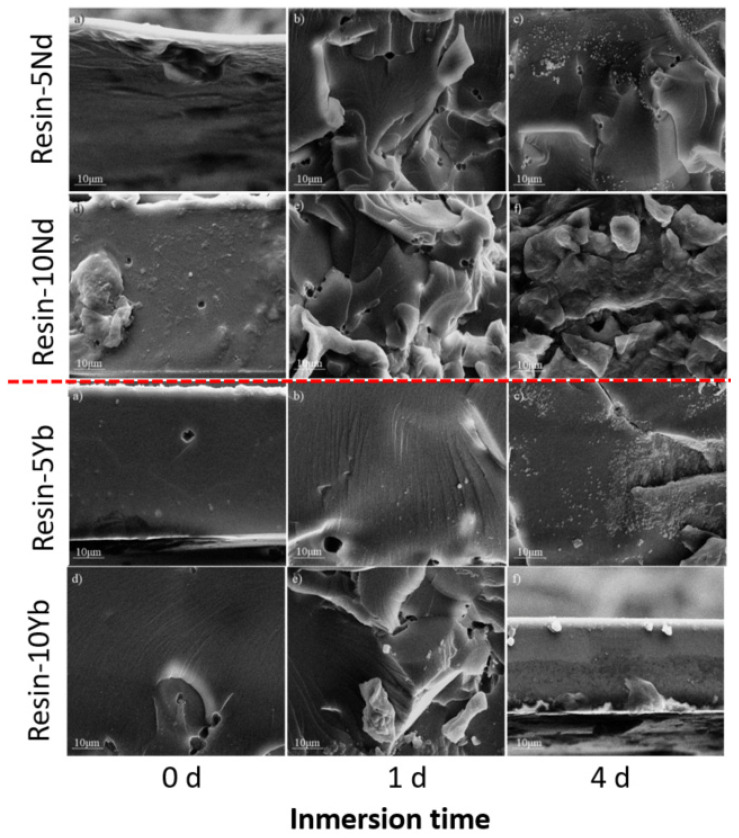
SEM images of the resin-xLn samples before and after immersion in 5 wt. % NaCl for 1 and 4 days. The scale is 10 µm.

**Figure 5:**
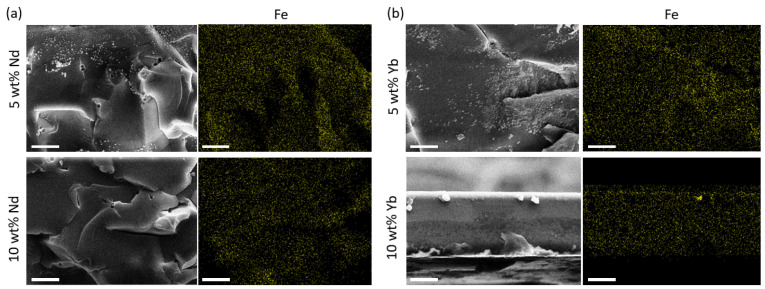
SEM images and corresponding EDS elemental mapping for the distribution of Fe elements in the resin-xLn after immersion in 5 wt. % NaCl for 4 days: (**a**) resin-5Nd (up) and resin-10Nd (down), and (**b**) resin-5Yb (up) and resin-10Yb (down). Scale bar: 10 µm.

Figure 4 shows the SEM images of the samples doped with 10 wt. % of Na[Ln(tta)_4_] before and after immersion in 5 wt. % NaCl solution. A roughened morphology with defects was visible for the samples treated for 1 and 4 days, indicating that the saline solution caused significant corrosive attack on the samples. Additionally, the EDX results represented in Table 1 evidence the high content of C and O elements in all the samples. As expected, the samples contained elements of Na[Ln(tta)_4_] before and after immersion in the saline solution. Importantly, we noticed that small quantities of Fe were found in the samples exposed to the saline solution for 4 days. This was associated with the corrosion process of the carbon steel substrate. The findings are presented in Figure 5, with the EDX mapping images showing the homogeneous distribution of Fe elements.

**Figure 6:**
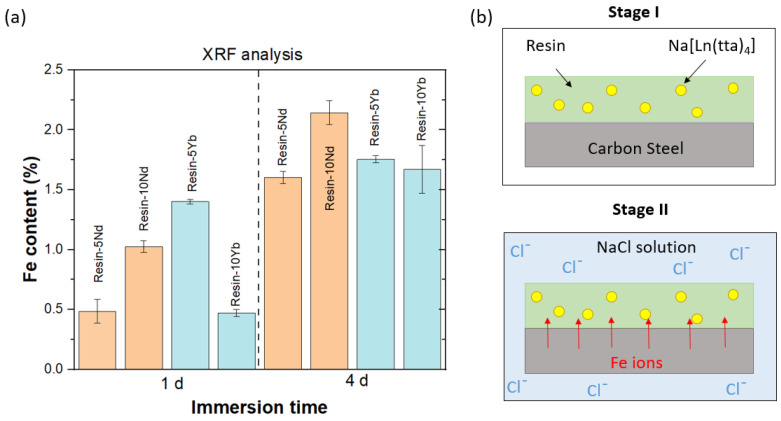
(**a**) A comparison of the Fe content values of the resin-xLn samples after 1 and 4 immersion days in 5 wt. % NaCl solution, as determined by XRF testing. (**b**) A schematic illustration of the system in the initial stage and after immersion in 5 wt. % NaCl solution.

The results obtained from the EDX analysis were complemented with an XRF test. Figure 6a and Table 1 depict the Fe contents found in the coatings after 1 and 4 days of immersion in the saline solution, showing that the Fe contents increased with the increasing immersion time, revealing the effect of the Cl^−^ ions on the carbon steel substrate. Thus, substrate corrosion leads to Fe diffusion within the coating, as illustrated in Figure 6b. In addition, it is important to draw attention to the differences found with respect to the above EDX analysis. Both XRF and EDX are analytical tools for semi-quantitative elemental analysis, and they have different detection limits. While EDX tends to be more effective for lighter elements, XRF shows the opposite trend. 

**Figure 7:**
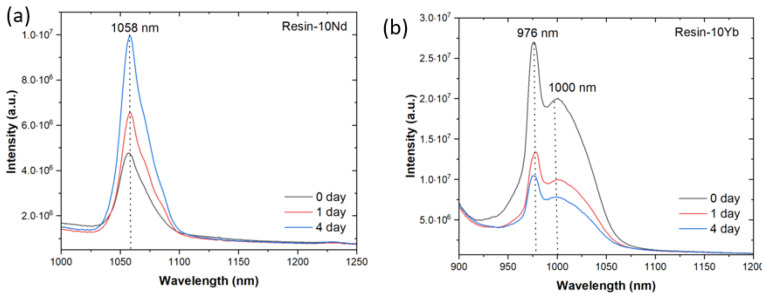
Emission spectra of the resin-10Ln samples, excited at 300 nm, before and after 1 and 4 immersion days in 5 wt. % NaCl solution: (**a**) Ln = Nd and (**b**) Ln = Yb.

Finally, the functional performance of the systems was evaluated. The PL spectra were measured to determine the sensing efficiency of the coatings against steel corrosion within the saline solution. Figure 7 show the PL changes of the resin with 10 wt. % of Na[Ln(tta)_4_] for Ln = Nd^3+^ and Yb^3+^, respectively, as a function of exposure time to a saline medium. In the case of the sample doped with Nd^3+^ (Figure 7a), one emission band, peaking at 1058 nm, is observed. In contrast, in the case of the resin incorporating Yb^3+^ (Figure 7b), two distinct peaks are identified at 976 nm (sharp) and around 1000 nm (broad). These results are in agreement with the characteristic emission wavelengths of these lanthanide ions, with the 1058 and 976 nm peaks corresponding to the characteristic Nd^3+^ (^4^F_3/2_→^4^I_11/2_) and Yb^3+^ (^2^F_5/2_→^2^F_7/2_) intra-4f transitions in the near-infrared (NIR) spectral range, respectively [31]. 

With respect to the effect of the immersion time on the PL spectra, the first observation is that the intensity of the PL emission gradually increases in the case of the sample containing Nd^3+^ ions (Figure 7a), whereas a decrease in intensity is observed for the sample doped with Yb^3+^ (Figure 7b), showing the significant differences of both ions upon exposure to a corrosive medium. Those differences are related to the specific characteristics of the ions, including the luminescence lifetime, cross-sectional energy gap, and quantum efficiency, among others, as well as their different interactions with the host matrix. For example, Nd^3+^ has a large energy gap between the most dominantly emissive ^4^F_3/2_ spin orbit level and the lower ^4^I_5/2_ level, while Yb^3+^ possesses a large absorption cross section of around 980 nm. In fact, this effect is also reported in related studies, regarding emission variations in lanthanide compounds, for sensing applications [32,33,34]. The values of the variation in the PL intensity as a function of time have also been determined. The PL intensity of Nd^3+^ increases up to around 27% after 1 day and 52% after 4 days. Meanwhile, for the 976 nm emission transition of Yb^3+^, this percentage difference is reduced in 50.3% after 1 day and 61.13% after 4 days. This result is in accordance with the observed reduction in the 1000 nm band, with variations of up to 50.13% and 60.95% found after 1 and 4 days, respectively. 

The obtained PL emission intensity results suggest that resins containing a functional luminescent lanthanide complex salt can potentially interact with the Fe compound and change the PL properties. They absorb energy and transfer it to the lanthanide metal to enhance the luminescence intensity or quench the luminescent signal. Several works have attempted to develop luminescent Ln-based sensors for Fe detection. For example, Orcutt et al. [35] developed a lanthanide-based chemosensor for bioavailable Fe^3+^. The studies showed that the displacement of the lanthanide-associated bands with Fe (III) is achieved in organic solvents. Yu et al. [36] demonstrated that several lanthanide metal–organic framework (MOF) compounds present an ultra-sensitive luminescent sensing response towards Fe (III). In addition, Fan et al. [37] studied the application of lanthanide compounds as fluorescent sensors for Fe (III). The results showed that samples are highly selective and sensitive luminescence probes for Fe (III) ions. Focusing on the luminescent sensing mechanism in these previous studies, it is related to variations in the charge transfer and energy transfer transitions. Despite these advances in designing sensors for iron detection, there are still challenges to overcome, such as improving the understanding the energy level modifications and electron–photon coupling parameters, which can provide deeper insights about luminescence sensing mechanisms. Therefore, it is necessary to support experimental analysis with theoretical methods, such as frontier molecular orbital analysis or quantum chemical approaches.

Thus, the variation in the PL emission spectra of the coatings in the presence of Fe ions allows us to monitor the evolution of corrosion over time. Therefore, they work as corrosion sensing coatings. Further, the sensing layer can be placed directly on the corroding surface of on top of another protective layer in a multilayer protective sensing system.

## 4. Conclusions

This study focused on the development of sustainable coatings with the ability of sensing corrosion. Specifically, the composite coatings fabricated by the combination of luminescent salts and the soybean vegetable matrix were applied to carbon steel substrates, and the system was stored in a saline medium. The morphological and structural features of samples were studied and the results were confirmed by the effective corrosion of carbon steel in the saline solution. Importantly, the functional sensing capability was evaluated though the variation in the PL of the coatings. The PL results show that the type of filler plays a profound role in the sensing response, the Nd-based salt showing a better performance than the Yb-based salt. Our results demonstrate that composites based on eco-friendly and sustainable materials show great potential to be used in sensing-based devices for corrosive environments. 

## Figures and Tables

**Table 1:** Elemental compositions of the resin-xLn as a function of immersion time (*t_i_*) in the NaCl solution as calculated by EDX.

Ln	*x*	*t_i_* (day)	Elemental Composition (wt. %)								
			C	O	Cl	Ln	F	Na	K	Ca	Fe
**Nd**	**5**	0	70.02	26.19	0.99	0.49	0.84	0.46	0.74	0.25	0
		1	67.27	29.49	0.45	0.69	1.76	0.34	0	0	0
		4	65.85	31.04	0.51	0.38	1.09	0.5	0	0	0.62
	10	0	63.36	30.24	1.07	1.02	3.03	0.73	0.37	0.19	0
		1	60.17	34.1	0.74	0.99	3.04	0.45	0.41	0.1	0
		4	64.95	29.82	0.37	1.12	2.8	0.32	0	0	0.62
Yb	5	0	62.48	34.25	0.17	0.53	1.82	0.19	0.07	0.08	0
		1	67.32	29.2	0.23	0.65	1.4	0	0	0	0
		4	60.15	28.19	4.13	0.57	0.88	4.53	0.08	0	0.96
	10	0	66.04	27.62	0.2	1.58	2.67	0.22	0	0	0
		1	57.45	34.54	1.49	1.43	2.87	0.98	0.59	0.65	0
		4	60.67	32.39	0.37	1.39	2.71	0.35	0.27	0.7	0.18

## Data Availability

Data are contained within the article.
